# A Qualitative Study of Factors Influencing Implementation of Tobacco Control in Pediatric Practices

**DOI:** 10.1155/2022/4156982

**Published:** 2022-05-05

**Authors:** Emara Nabi-Burza, Jonathan P. Winickoff, Jeremy E. Drehmer, Maurice P. Zeegers, Bethany Hipple Walters

**Affiliations:** ^1^Massachusetts General Hospital for Children, Division of General Academic Pediatrics, Boston, MA, USA; ^2^Massachusetts General Hospital, Tobacco Research and Treatment Center, Boston, MA, USA; ^3^Harvard Medical School, Boston, MA, USA; ^4^American Academy of Pediatrics, Julius B. Richmond Center of Excellence, Itasca, IL, USA; ^5^Nutrition and Translational Research in Metabolism (School NUTRIM), Maastricht University, Maastricht, Netherlands; ^6^Care and Public Health Research Institute (School CAPHRI), Maastricht University, Maastricht, Netherlands

## Abstract

**Introduction:**

Clinical effort against secondhand smoke exposure (CEASE) is an evidence-based intervention that prepares child healthcare clinicians and staff with the knowledge, skills, and resources needed to ask family members about tobacco use, provide brief counseling and medication assistance, and refer to free cessation services.

**Aim:**

This study sought to identify factors that influenced the implementation of CEASE in five pediatric intervention practices in five states that participated in a cluster randomized clinical trial of the CEASE intervention.

**Methods:**

Guided by questions from the consolidated framework for implementation research (CFIR) interview guide, semistructured qualitative interviews were conducted with 11 clinicians and practice staff from five intervention practices after the practices had implemented CEASE for two years. Interviews were conducted by a trained qualitative researcher, recorded with permission, and transcribed verbatim. An interview codebook was inductively developed; two researchers used the codebook to code data. After coding, data was analyzed to identify factors, as described by the CFIR domains that influenced the implementation of CEASE.

**Results:**

The implementation of CEASE in practices was influenced by the adaptability and complexity of the intervention, the needs of patients and their families, the resources available to practices to support the implementation of CEASE, other competing priorities at the practices, the cultures of practices, and clinicians' and office staffs' knowledge and beliefs about family-centered tobacco control.

**Conclusion:**

Identifying and influencing certain critical factors guided by information gathered through interviews may help improve implementation and sustainability of family-centered tobacco control interventions in the future. Trial Registration: ClinicalTrials.gov Identifier: NCT01882348.

## 1. Introduction

National Health and Nutrition Examination Survey (NHANES) data shows that 35.4% of children aged 3–17 years in 2013-2016 were exposed to tobacco smoke in the US [1], despite well-documented risks from tobacco smoke exposure to children's health [2, 3]. Pediatric clinicians are uniquely positioned to address family tobacco use and reduce children's exposure to tobacco smoke by helping families quit smoking [4, 5]. While clinical practice guidelines recommend that clinicians and staff in child healthcare settings, such as pediatric offices, screen families for tobacco use and provide guidance to reduce tobacco use and exposure [6–8], few pediatric practices routinely ask about tobacco use and offer evidence-based cessation assistance [9, 10].

### 1.1. Clinical Effort against Secondhand Smoke Exposure (CEASE)

Research has shown that many child healthcare clinicians and staff lack the skills and confidence needed to address family tobacco use and exposure, revealing gaps in knowledge and in practice [11]. The clinical effort against secondhand smoke exposure (CEASE) intervention was developed to address such gaps [11]. The development of CEASE was and continues to be informed by evidence-based tobacco control guidelines [7, 12], smoking cessation strategies and tools [13, 14], and insights from tobacco control specialists, public health professionals, and clinicians [15–17].

Through CEASE, child healthcare clinicians and staff are trained to address family smoking and family tobacco smoke exposure. The CEASE capacity building efforts are centered around two training calls (a peer-to-peer training call for practice champions and a training call for the whole office) [18], with opportunities for additional training through an online CME program in tobacco control [19] and the American Academy of Pediatrics' Maintenance of Certification online course in tobacco control [18, 20].

In line with ask, assist, and refer [21], the core components of CEASE consist of screening families for tobacco using an iPad-based previsit screener and assisting with cessation by providing evidence-based tobacco cessation treatment and referral to free cessation support services to those who smoke. The previsit screener, which is used exclusively for the intervention, is given to the adult family member accompanying the child at the visit. The adult family member, commonly a parent or legal guardian (hereafter referred to as *parent*), completes the previsit screener before the parent and child are seen by the clinician; this often happens during check-in at the front desk, in the exam room, or at another previsit moment. The previsit screener identifies families exposed to tobacco smoke with this question: “Does any member of your household use any form of tobacco?” If the parent indicates that no members of the household use tobacco, no further questions are asked. Parents who report having a household tobacco user are asked additional questions. These questions include information about their child's name, relationship to the child, and the parent's own tobacco use status. If the parent is a current smoker, the screener is programmed to ask them about their interest in nicotine replacement therapy (NRT) patch and gum prescriptions and referral to the free state quitline and SmokeFreeTXT program [22]. After the parent who smokes completes all questions, a member of the front desk staff gives the parent preprinted NRT prescriptions. If the parent indicates on the screener that he or she would like to be referred to the quitline, the front desk staff are prompted to give the parent a tobacco quitline enrollment form. When available from the state's tobacco quitline, the previsit screener includes information about when to expect a call from the quitline and/or how the phone number would likely appear on their caller ID.

CEASE has been shown to be effective at helping parents quit smoking [10]. The economic evaluation of the CEASE intervention showed an incremental cost-effectiveness ratio of $1132 per quit [23]. However, less is known about the factors that influence the implementation of CEASE in pediatric office settings. Understanding the factors that influence the implementation of CEASE is crucial for the scale-up, sustainability, and dissemination of evidence-based family tobacco cessation interventions in child healthcare settings.

## 2. Methods

As part of a hybrid effectiveness/implementation study of CEASE in five intervention pediatric practices in five states (OH, NC, TN, VA, and IN) (ClinicalTrials.gov identifier: NCT01882348) [10], interviews were conducted with pediatric clinicians and staff gain insight into the factors that influenced the implementation of CEASE in study practices.

### 2.1. Ethical Approval and Consent

The study protocol was approved by the Institutional Review Boards at the American Academy of Pediatrics, Massachusetts General Hospital, and individual practice IRBs when required. In addition, all respondents were consented before data collection, and verbal permission was given to record the interview.

### 2.2. Design

Practices were recruited into the hybrid effectiveness/implementation study through the American Academy of Pediatrics. Practices were eligible if they had parent smoking prevalence >15%, average patient flow >50 families/day, >four full-time clinicians, and used an electronic health record (EHR). Eligible practices that expressed interest conducted three-day practice population surveys (PPS) to confirm parent smoking and patient flow rates.

As part of the study, intervention practices were asked to identify a pediatrician to serve as a practice champion, who would support the implementation of CEASE in their practice. Also, a member of the office staff, such as an office manager, was asked to serve as a coordinator for the CEASE study and the implementation of CEASE in their practices. The practice champion and the coordinator at each practice were asked to participate in interviews about implementation of CEASE in their practices.

Interviews were conducted two years after the start of CEASE implementation, which is defined as two years after clinicians and staff were trained in the intervention and after practices began using the iPad-based previsit screener to screen families for tobacco use and exposure. The semistructured interviews were conducted via telephone between November 2017 and January 2018 by a PhD-level researcher who was a part of the CEASE research team (BHW).

### 2.3. The Use of the Consolidated Framework for Implementation Research

The consolidated framework for implementation research (CFIR) is a comprehensive, theory-informed, and adaptable implementation research framework consisting of five domains that have been shown to shape the implementation of interventions; these domains are intervention characteristics, the outer setting, the inner setting, the characteristics of individuals, and the process of implementation [24, 25]. Each of these domains consists of a variety of subdomains, which help provide further details for each of the domains. In the CEASE study, the CFIR was used to develop the interview guide and to analyze data collected through interviews.

### 2.4. Interview Guide

Clinicians and staff were interviewed using questions from an interview guide, which consisted of tailored questions from the CFIR interview guide [26, 27] and questions specific to the CEASE intervention. The interview guide was reviewed by the study's steering committee and further improved based on feedback from an external qualitative researcher who reviewed it for potential leading questions, relevance, and clarity.

### 2.5. Interview Process

The phone interviews lasted between 45 and 60 minutes. In the preinterview briefing, respondents were encouraged to be open and honest, there were no right or wrong answers, the focus of the interview was to learn about their experiences with implementing CEASE, and respondents had the right to stop the interview at any time or to skip questions. The respondents were assured that the data would be anonymized.

### 2.6. Data Analysis

The interview recordings were transcribed verbatim using an external service. The transcriptions were read closely and anonymized by BHW. She then shared the cleaned transcripts with ENB. Both coders (BHW and ENB) closely read all transcripts before coding.

Once the transcripts had been cleaned and read, BHW began inductively coding five transcripts. The codes that were uncovered during this initial coding process were used to develop the codebook. The codebook included key terms (codes), definitions of the codes, inclusion and exclusion criteria for each code, and an example quote that was representative of each code. After this initial development, the codebook was shared with the second coder (ENB), who coded a sample of the transcripts and added to the codebook. The revised codebook was then reviewed by both coders; both coders then met to discuss any questions about the codebook. After this meeting, the codebook was approved and finalized. The final codebook contained 33 codes. The final codebook was used as a guide for coding all transcripts.

The transcripts were coded independently by the two investigators. Coding was done in Word. Each code was documented onto its respective code page. The coder copied relevant quotes from the transcript into the relevant code page. If a quote met the inclusion criteria for two codes, the quote was copied into the relevant code pages for both codes. This was done for all transcripts. After all of the transcripts were coded, the coders had a series of five- to six-hour-long meetings in which they compared their code pages for each of the codes. During these meetings, the coders discussed any differences in coding and resolved them based on the contents of the quote, each code's definition, each code's inclusion criteria, and each code's exclusion criteria. At the end of these meetings, all coded data had been reviewed and agreed upon by both coders, resulting in a final set of coded data.

The coded data was analyzed using a thematic approach [28]. Themes and included categories were organized into a thematic framework. Major themes were mapped to the domains of the CFIR [24, 29].  Table 1 presents the CFIR domains with definitions, relevant constructs with definitions, and major themes from the data mapped to the domains.

## 3. Results

The interviews were conducted with 11 respondents from the five intervention practices. Of these, four respondents were MDs, one was a receptionist, five were office managers, and one was a practice nurse. In one practice, the office manager was also a clinical provider (nurse) at the practice and was the most involved in the intervention implementation, so she was the only one interviewed at that practice.

### 3.1. Intervention Characteristics that Influence CEASE Implementation

The CFIR defines intervention characteristics as “key attributes of interventions that influence the success of implementation. The core components and characteristics of CEASE included screening for tobacco use using the iPad, referring parents to the free state smoking cessation telephone support service using a fax-to-quit form, and prescribing NRT to parents using pre-printed prescriptions.” These “key attributes” [24] of CEASE shaped, in part, how practices implemented the CEASE intervention. Interview data provided insight into practice implementation and the adaptability of CEASE, as well as the complexity of CEASE.

#### 3.1.1. The Adaptability of the CEASE Intervention

The adaptability (the degree to which an intervention can be adapted, tailored, refined, or reinvented to meet local needs) of the CEASE intervention was a recurring theme in the interviews. During trainings and communication with the CEASE study team, practices were encouraged to tailor the adaptable periphery of CEASE [24], such as health education materials and the use of the iPad screener, to work within existing practice workflows and to meet the needs of the practice's patients and their families.

Respondents reported adapting CEASE to work within their practice's existing workflow and processes. The practice 2 office manager stated that “we looked at our processes and changed things, tweaked it a little bit… we were able to kind of overcome a lot of those things and get back to this being part of the workflow instead of this added thing.” Other practices reported changing the process of using the iPad to screen parents for tobacco use and exposure; the practice 1 pediatrician explained “instead of doing [the iPad] at all visits, we did it only at well-child visits.”

Adapting CEASE included tailoring the provided parental health education materials, as well as creating additional, practice-specific health education materials to help parents quit smoking. The practice 1 pediatrician noted that “we put the handouts together about how to use patches correctly, and we also put in the information for the quitline and we put information in [about] tobacco classes, free classes from (inaudible) hospital that were offered so that you get free nicotine patches. So we tried to, ourselves, do some education for our patients.”

#### 3.1.2. The Complexity of the CEASE Intervention

During the interviews, many pediatricians and office staff shared that using iPads to routinely screen for parental tobacco use was a complex aspect of the CEASE intervention, as it could involve the use of a new tool (the iPad), a different or longer check-in process for families, additional tasks for staff, and/or resistance from parents. Some of the complexity of using the iPad screener was related to how families reacted to the intervention; the office manager at practice 4 mentioned that “I think the only issue we had was people taking it over and over again that weren't interested in it.” In other cases, the complexity of the screener was related to the process of using it routinely in practice. The pediatrician at practice 4 noted that “handing out the iPad at the front desk, I think, was not an easy thing to implement.”

While using the iPads may have been complex for practices, some respondents noted their pride in being able to screen most of their families for tobacco use. The practice 3 office manager noted that “some days we were really busy, and we were trying to catch them with all the iPads, and it maybe slowed down a little bit. But at the end, we were happy that we could manage to do it with every family.”

### 3.2. The Role of Outer Setting in the Implementation of CEASE

The implementation of the CEASE intervention was shaped by factors outside of the pediatric practices. The factors included the needs of patients and their families, external policies governing the care provided by practices, and external incentives for clinicians and office staff.

#### 3.2.1. The Needs and Resources of Patients and Their Families

Interview respondents noted that their communities and their patient populations were in need of an intervention to address tobacco use and children's exposure of tobacco smoke. The CEASE intervention was seen as a potential way of addressing the needs of families with regard to tobacco use and exposure. The practice 2 office manager explained that “I think (it was needed) within our population just because it's largely tobacco using. I think it was definitely needed in our area for sure.” Further, a pediatrician in practice 3 noted that “we have 30, 20-25 percent of patients per day (with) parents that smoke so definitely we get secondhand and thirdhand smoke, (which) we can decrease. And it's going to definitely going to help the children, their sickness, their getting repeatedly sick, those asthma patients.” The perceived need for such an intervention may have influenced how, to what extent, and with which families practices used the CEASE intervention.

Many of the practices in the study served high need, low-income families with high rates of tobacco use; during the interviews, practice staff and clinicians reported that their practices had high number of patients insured through Medicaid, which is commonly associated with higher levels of tobacco use [30]. The practice 5 office manager stated that “at least 50 percent of our patients are Medicaid patients.” While many of the children seen at the practices were insured through Medicaid, interview respondents noted that many parents lacked health insurance for themselves. The practice 4 pediatrician noted that “it was eye-opening to me to realize how many of our parents do not have insurance.” In addition to many parents having no insurance, respondents also noted that parents did not have the financial resources to pay for cessation medication out-of-pocket. The practice 4 office manager explained that “there were people that wanted to quit; they just really couldn't afford the patches or the gum, and they didn't have any insurance.”

#### 3.2.2. External Policies and Incentives

Interview respondents noted that it could be difficult for parents to access smoking cessation medication due to financial constraints. However, at various time during the implementation of CEASE, some of the state quitlines offered free nicotine replacement therapy to those enrolled in quitline services. This free NRT served as an external incentive for the practices to enroll parents in the quitline; the practice 2 office manager noted that “sometimes they (quitline) would offer two free weeks of NRT for anyone no matter what their insurance status…. So that was a great support.” Practices were motivated and indirectly incentivized to enroll parents in the quitline by this external policy of free NRT.

### 3.3. The Role of the Inner Setting in the Implementation of CEASE

Respondents described how the structure of the practices, the context and culture of the practices, the organizational incentives for implementing CEASE, and the other programs and care provided by the practices influenced the implementation of CEASE in their practices.

#### 3.3.1. Implementation Climate: Organizational Incentives

Organizational incentives to implement an intervention include tangible and intangible incentives, such as increased opportunities for payment and potential for advancement or professional development [24].

During the interviews, a few respondents noted that payment (or the hope of payment) from insurance companies helped their practice implement CEASE. The practice 4 office manager explained that “it's not a large amount by any means, the ones [insurance providers] that do pay on it. But it was just that extra incentive to get $10 to $20 a visit extra because you spent some time counseling with the patient… So that was a pretty big incentive, and like I said, when we figured that out that was when the doctors, kind of, took ownership of it because of the financial incentive as well.” The additional funds served to support implementation and motivate some pediatricians to spend more time talking about smoking cessation with parents. However, many practices were not able to successfully bill insurance companies for the services that they provided, even when payment for those services was legally obligated [31].

Not all incentives to implement CEASE were directly financial. As one respondent noted, the Maintenance of Certification (MOC) course offered through CEASE was an incentive in and of itself. While the MOC course does have a monetary value, the added value for participants was seen to be in the overlap between the CEASE training and the MOC course; as part of CEASE, pediatricians were already learning and practicing much of the content of the MOC. The practice 4 pediatrician stated that “it was for MOC credit and all of us are scrambling for that MOC 4 credit because MOC 4 is the hardest to get… So that was a good incentive to get everybody.” The course served as both a resource and an incentive to implement CEASE. As MOC credits are required to maintain certification, this aspect of CEASE can be seen as an indirect “organizational incentive” [29].

#### 3.3.2. Implementation Climate: Relative Priority

Many respondents described facing conflicting priorities and demands on their time, which impacted the implementation of CEASE. The practice 3 pediatrician stated “we're doing a study on asthma. We're doing a study on digestion. We're doing a study on breastfeeding. And when you do so many things, time was a constant (problem),” while the practice 1 pediatrician noted that “we've got to do all the regulatory stuff that is being asked of us in well-visits and sick-visits. So, adding this extra CEASE component really was kind of a juggling act for us, if you can imagine that.” The office manager at practice 4 noted that “Probably the biggest obstacle for us was the amount of presumed work it was to get the iPad component embedded in what we were doing because when patients come to the front desk to check in they're already confronted with a variety of things they have to fill in each time, whether it's the developmental screening, or changes in their insurance information, or whatever, or verification of those things.” Many respondents noted that sometimes they had other priority tasks to complete which were seen as a barrier to the implementation of CEASE.

#### 3.3.3. Culture

The culture of an organization—“the norms, values, and basic assumptions” [24]—influences how an intervention is implemented. The respondents noted that the alignment of the goals of the intervention with the organizational culture affected the intervention implementation in a positive way. This was reflected in practice 1 office manager's quote, “I think we're very involved in the community and making sure that our population and community is healthy …. [I] feel like ethically, that's what we have to do”; the practice 1 office manager went on to say that “we were able to make a positive impact on not only the health of our parents, but also our children that we see. So I feel like culturally, it [CEASE] fit right in with what we do.” Interventions that align with the overarching culture of an organization tend to be more successfully implemented [29].

### 3.4. Characteristics of Individuals

During the interviews, pediatricians and office staff described how their personal beliefs and knowledge about parental tobacco use and the tobacco smoke exposure of children motivated them to implement CEASE. They also described the ways in which their knowledge and beliefs shaped the way that they worked with other staff members to implement CEASE in their practices.

#### 3.4.1. Knowledge and Beliefs about Family-Centered Tobacco Control

Many respondents described their belief that addressing parental tobacco use and the tobacco smoke exposure of children was an important responsibility of child healthcare clinicians. The practice 1 pediatrician stated that “I feel very, very strongly that there are certain things that we should be doing as healthcare providers to keep certain general healthcare parameters high on the radar because if we don't tell our patients that we're concerned and think these things are important, they're not going to see that as an important thing. So, if we don't have smoking as something that we talk about, to try and educate and to let them know that we think that this is an important issue to address, just like obesity and healthy lifestyle -- if we're not actively promoting those things, then I think we're not fulfilling our mission.” The implementation of CEASE, then, helped the pediatrician and their practice to fulfil their sense of mission.

Respondents described how CEASE gave them the motivation, tools, and knowledge needed to address parental tobacco use. The practice 5 pediatrician stated that “I am proud that we've actually talked -- because it did get us to discuss more of secondhand smoke for kids, and thirdhand smoke, and what that meant. I am proud that we actually did talk to parents about that… So, I think that was good because it got awareness out there, so parents actually know that we were serious.” Through CEASE, clinicians were able to share their knowledge to increase awareness of second- and thirdhand smoke while also sharing their beliefs in the seriousness of parental smoking and the tobacco smoke exposure of children.

### 3.5. Process of Implementing CEASE

Through the interviews, pediatricians and office staff were asked to describe how the practice is prepared for implementing CEASE and the workflows and step-by-step actions conducted by different staff members used to implement CEASE in their practices and to share insight into how staff worked together to implement CEASE.

#### 3.5.1. Preparing for and Engaging with CEASE

Respondents described how working together, such as brainstorming as a team, was a part of their practices' implementation process. The practice 1 receptionist said that “we actually had a meeting -- a staff meeting with the nursing staff, clerical staff, and the providers, and I think we were just brainstorming ways of how to make this process run smoother.”

In addition to brainstorming and planning at the beginning of the CEASE project, pediatricians and office staff described how they adapted the workflow and different staff roles over time, engaging with one another to improve the process of implementing CEASE in their practices; the practice 4 office manager noted that “In the beginning, I think, the doctors thought it would be more of a nurse-and-reception thing and it -- it wasn't going so well, so we switched up, and they took a lot more ownership in it, I guess, and that was when we saw more success.” Engaging with CEASE—“involving appropriate individuals in the implementation and use of the intervention”—was a process that evolved and adapted over time to meet the changing needs and realities of practices [24].

#### 3.5.2. Practice Champions for CEASE

During the interviews, pediatricians and office staff noted that having a supporter of the intervention helped in motivating other staff. The practice 3 office manager said that “somebody who is motivated be behind you and tell you, ‘Just keep going. We're not going to stop.'…. ‘How many do we want to do today? How many … people (are) coming to us today?' So, to have that motivation is very good,” while the practice 1 office manager noted that “he was up there talking about why it's important; I think it made people understand how, yes, this is something they need to do….. So I feel like those are things that helped encourage people to become more involved.”

## 4. Discussion

This qualitative study explores the factors that influenced the implementation of CEASE, an evidence-based family-centered tobacco control intervention, in five pediatric practices in the US. Interviews using questions from the consolidated framework for implementation research provided insight into the implementation of CEASE; the domains and subdomains of the CFIR provided a structure to understand the factors that may have influenced the implementation of the CEASE intervention in pediatric office settings. Through the interviews, pediatricians and staff indicated that the implementation of CEASE was shaped by

- the adaptability and the complexity of the intervention (CFIR domain: intervention characteristics)
- the needs and resources of the patients and their families (CFIR domain: outer setting)
- incentives for implementing CEASE and practice's culture (CFIR domain: inner setting)
- knowledge and beliefs about family-centered tobacco control (CFIR domain: characteristics of individuals)
- engaging staff with CEASE and practice champions for CEASE (CFIR domain: process) [24, 25]

### 4.1. Perceived Complexity of (Implementing) CEASE

As described by the CFIR, interventions (and implementing these interventions) can be understood as complex when they have both core components and an adaptable periphery—elements of the intervention and of the implementation of the intervention that can be adapted by staff at the practice to meet the practice's needs [29, 32]. Interventions are also considered complex when they have a number of interacting components and involve (potentially) difficult changes to behaviors and activities by those conducting the intervention [33]. In their guidance on how to evaluate complex interventions, G. Moore et al. noted that programs to help people quit smoking are often complex [34]. Using these conceptualizations, the CEASE intervention and its implementation can be seen as complex. Data from the interviews revealed that having the ability and flexibility to adapt components of CEASE and its implementation was seen as an opportunity to adapt CEASE to the practice, using an iPad to routinely screen for tobacco use, and exposure was often viewed as difficult and disruptive. This complexity may influence how CEASE is scaled up to nonresearch practices, as well as to what extent practices can engage with and sustain CEASE over time.

### 4.2. Needs and Resources of Patients and Their Families

The implementation of CEASE was shaped by factors outside of the pediatric clinicians and office staff. The overall effect of factors in the outer setting is similar to what Pettigrew et al. [35] called the “receptive context for organizational change,” which emphasizes identifying the external factors that that influence intervention implementation and the importance of interventions to adapt to these factors. Implementation can be positively influenced by the degree to which an intervention meets the perceived needs of patients and their families [36]. Studies have also shown that smokers with lower incomes are less likely to use evidence-based smoking cessation treatments like pharmacotherapy than smokers with higher incomes [37, 38]. Although Medicaid covers NRT patch and gum [31], many insurance companies do not cover it, and many parents do not have any insurance. While CEASE has been designed to use existing evidence-based counseling programs and covered medications to help parents quit smoking, this relies on the programs and medications being easily and feasibly available to parents. Without enforcement of required medication coverage at the insurance company level and availability of free tobacco quitline and texting programs, it may be difficult for parents to access the treatments prescribed by pediatricians as part of the CEASE intervention.

### 4.3. Incentives for Implementing CEASE and the Practice's Culture

The inner setting of practices also played a key role in the implementation of CEASE. Financial incentives, such as receiving payments from insurance companies for the time spent in addressing the tobacco smoke exposure of children, was seen as an incentive by some respondents. Other nonfinancial incentives included the opportunity to earn MOC credits required to maintain certification and a CME-awarding course on tobacco control. These findings are consistent with the literature that suggests that incentives, including financial incentives and performance evaluations, positively influenced intervention implementation [39].

### 4.4. Knowledge and Beliefs about Family-Centered Tobacco Control

The respondents' knowledge of CEASE and beliefs about tobacco use influenced its implementation. These beliefs are important to understand at the individual and practice level to assess quality of implementation and prospects for sustainability [40]. Adequate knowledge of the intervention affects the adoption of the intervention, and often, opinions based on personal beliefs and experiences are convincing and help to generate enthusiasm about the intervention [41].

### 4.5. Engaging Staff with CEASE and Practice Champions for CEASE

The interview data showed that having individuals who are internally motivated to support implementation influenced how the intervention was implemented in their practices as they served as a driver of motivation. Engaging staff in a meaningful problem-solving manner is a critical element to transform patient care [42]. Data also showed that engaging staff and reflecting on the reasons for doing the intervention was key to implementation. Dedicating time for reflecting or debriefing during and after implementation was one way to promote shared learning and motivation along the way [43].

Table 2 presents the main challenges faced that were learned from this qualitative study and the implications and improvements for dissemination and sustainability of the intervention.

### 4.6. Limitations

The small sample size may limit generalizability of results, though the themes identified were consistent across five practices and enhance the likelihood that the findings are not unique to a specific pediatric practice [44]. The results are limited to respondents who agreed to take part in interviews, which could have resulted in selection bias. Respondents other than those interviewed may have had different responses than those reported here and may not be representative of other pediatric clinics. However, we aimed to interview both clinical and administrative staff from each practice to get the overall picture of intervention implementation. Since the interviews were conducted with respondents from five pediatric practices in five states across the US, the diversity of the sample gives us greater confidence that the findings of this study may be applicable and potentially transferable to other US pediatric clinics [44].

## 5. Conclusion

This study examined the implementation of an evidence-based tobacco control intervention, CEASE in pediatric outpatient settings. We identified certain factors that may help improve implementation and sustainability of tobacco control interventions in the future. Findings from this paper emphasize the importance of intervention characteristics (more adaptable and less complex), inner setting (incentives for implementing CEASE and practice's culture), outer setting (addressing the needs and resources of patients and their families), characteristics of individuals (knowledge and beliefs about the intervention), and the process of implementing an intervention (engaging all staff roles with CEASE and having practice champions for CEASE). By attending to these factors, future tobacco control interventions will have the best possible chance of sustainable integration into routine care delivery and enhanced likelihood of effective dissemination.

## Figures and Tables

**Table 1:** Major study themes mapped to the CFIR domains [24, 25].

CFIR domains and definition	Constructs and definition	Themes
Intervention characteristics (key attributes of an intervention that may impact implementation success)	(i) Complexity of CEASE (perceived difficulty of the intervention, reflected by duration, scope, radicalness, disruptiveness, centrality, and intricacy and number of steps required to implement) (ii) Adaptability of CEASE (the degree to which an intervention can be adapted, tailored, refined, or reinvented to meet local needs)	(i) Screening all families at every visit for tobacco use with an iPad (ii) Ability to adapt or make changes to CEASE
Outer setting (includes the features of the external context or environment that might influence implementation)	(i) Patient and family needs and resources (the extent to which patient needs, as well as barriers and facilitators to meet those needs, are accurately known and prioritized by the organization)	Patients and their families: (i) Need for tobacco control (ii) Patient population characteristics (iii) Parents barriers to CEASE (iv) Parents response to CEASE External cessation support: (i) Access and coverage for NRT (i) Free help and resources from the quitline
Inner setting(includes features of the implementing organization that might influence implementation)	(i) Implementation climate (ii) Organizational incentives (extrinsic incentives such as goal-sharing awards, performance reviews, promotions, and raises in salary, and less tangible incentives such as increased stature or respect) (iii) Relative priorities (individuals' shared perception of the importance of the implementation within the organization) (iv) Culture (norms, values, and basic assumptions of a given organization)	CEASE implementation in the office (i) Extent to which the practice was able to bill for these services (ii) CEASE vs. other priorities (iii) MOC credits as an incentive Organizational culture (i) Culture of the practice
Characteristics of individuals (characteristics of individuals involved in implementation that might influence implementation)	(i) Knowledge and beliefs about the intervention (individuals' attitudes toward and value placed on the intervention as well as familiarity with facts, truths, and principles related to the intervention)	CEASE and staff (i) Second- and thirdhand smoke beliefs and knowledge (ii) Beliefs and motivation of the staff to address tobacco use in families (iii) Feeling proud for helping smoking family members quit
Process (includes strategies or tactics that might influence implementation)	(i) Engaging (attracting and involving appropriate individuals in the implementation and use of the intervention through a combined strategy of social marketing, education, role modeling, training, and other similar activities) (ii) CEASE champions (individuals who dedicate themselves to supporting, marketing, and “driving through” an [implementation] [101] (p. 182), overcoming indifference or resistance that the intervention may provoke in an organization)	(i) Engaging staff with CEASE (ii) CEASE champions as drivers or motivators for other staff members

**Table 2:** Challenges and implications for sustainability and disseminability of the intervention.

Challenges faced	Implications and improvements
The previsit screener identifies parents who smoke and offers them treatment but does not sign them up	The previsit screener could go a step further and automatically connect parents who smoke with resources to help them quit smoking to further reduce any burden on practice staff
The previsit screener was not integrated with other screeners and paper work leaving the front desk juggling multiple previsit tasks in different platforms at check-in	Integrate the previsit tobacco use screener with other previsit surveys in a single platform that would all populate the appropriate sections of the child's medical record
The previsit screener was offered too frequently	The previsit screener should screen all families once a year for tobacco use, following up with only those families that have a smoker at scheduled time intervals
Billing for services is a good financial incentive for the clinicians and practices but did not happen routinely	Billing for tobacco use counselling should be easy and automated in the child's medical record

## Data Availability

The deidentified dataset used to support the findings of this study are available from the corresponding author upon request.

## Additional Points

*Implications*. Healthcare organizations can improve the health of families by implementing an evidence-based family-centered tobacco control program, such as CEASE. This research showed that the implementation of CEASE was shaped by a variety of factors, including the complexity and adaptability of the program, the needs of patients and their families, the resources available to support implementation, conflicting priorities, and practice staff engagement. Strengthening or modifying factors that influence implementation may result in more successful implementation of evidence-based family-centered tobacco control programs.
